# Crop health management for food and nutritional security and soil health

**DOI:** 10.1007/s44297-026-00083-6

**Published:** 2026-08-06

**Authors:** Rattan Lal

**Affiliations:** CFAES Rattan, Lal Center for Carbon Management and Sequestration, The Ohio State University, Columbus, OH 43210 USA

**Keywords:** Human health, Environmental health, Soil health, One Health, Crop Health, Undernutrition, Malnutrition, Nutrition-sensitive agriculture

## Abstract

The concept of “crop health” is in accordance with the “one health” approach, which states that “health of soil, plants, animals, people, ecosystems, and the planetary processes is one and indivisible.” It specifically implies vigor and growth of crop plants, resilience against biotic and abiotic stresses, and ability to produce adequate amounts of nutritious and safe food while also protecting and restoring soil health and the environmental quality. The One Health approach is gaining momentum because of the widespread and persistent problems of undernutrition and malnutrition along with environmental degradation, especially in low-income countries in Sub-Saharan Africa, South Asia, and Latin America. The “Crop Health” approach is pertinent in the current era of escalating global warming, aggravating soil degradation, dwindling biodiversity, decreasing quality and renewability of water resources, the ever-growing problem of food and nutritional insecurity, and increasing risks of war and political instability. The strong interconnectivity of the nexus of soil, environment, and crop health is the driver of growing interest in the Crop Health approach and its management by improving soil health and environment quality.

## Introduction

The term “crop health” (CH) refers to overall physiologic attributes related to favorable rigor and growth of cultivated crops for producing a range of ecosystem services (ESs) for human wellbeing and nature conservancy. It focuses on economic and productivity outputs. In comparison, plant health refers to the overall physiologic wellbeing and resilience to biotic and abiotic stresses of any plants. Both crop and plant health are affected by soil health, which is the soil’s capacity to provide a range of ESs. There are specific indicators of CH that include, but are not limited to, resilience against biotic and abiotic stresses, vigor and genetic attributes, quality and quantity of agronomic productivity, and having a positive impact on the soil and environment. CH also indicates if a crop is free from infestation of pests and pathogens, has sufficient macronutrients and micronutrients, and is not susceptible to other growth-inhibiting factors, such as drought/flood syndrome and unfavorable (sub- or supra-optimal) soil temperature regimen. Thus, a healthy crop is expected to produce a crop yield of high nutritional quality and good safety standards for the ecoregion.

**Fig. 1:**
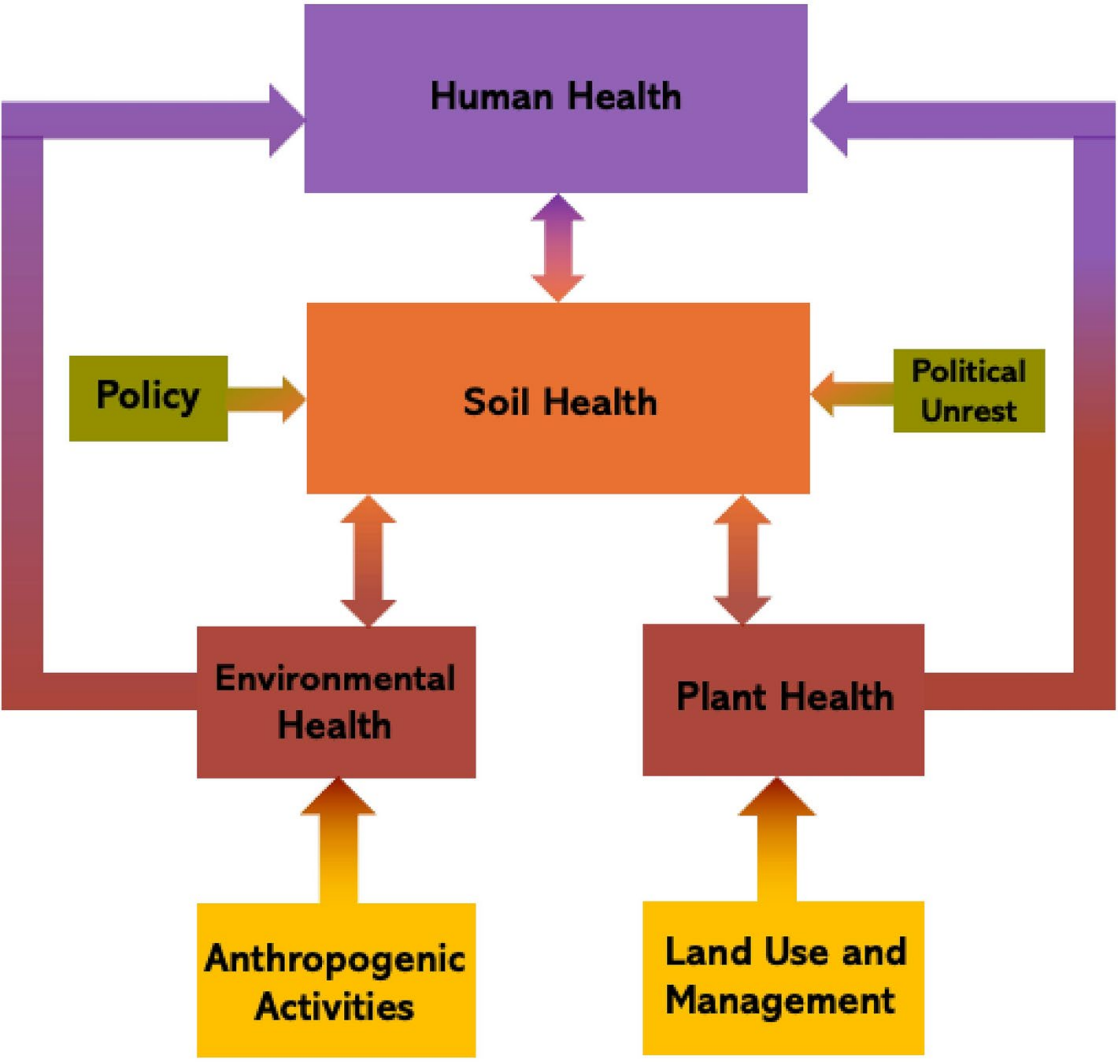
The “One Health” concept. Everything is connected to everything else. Anthropogenic activities, land use, and soil management affect plant and environmental health and the quality of food and forages produced. Soil health is also impacted by political and policy factors, which need to be pro-nature, pro-agriculture, and pro-farmer. All these factors interacting with each other moderate and strengthen the nexus of environment, soil, crop, and human health

A healthy crop must also support sustainable farming while protecting soil and environmental health by restoring the soil and the terrestrial carbon stock. Simply put, CH refers to the overall health of cultivated crops in relation to the production of adequate amounts of safe, nutritious food plus forages while restoring soil health and promoting adaptation to and mitigation of anthropogenic climate change (ACC). CH is in accordance with the “One Health” approach, which states that “health of soil, plants, animals, people, ecosystems, and the planetary processes is one and indivisible” (Fig. 1). There are several determinants of CH, including use of regenerative agriculture and agro-ecologic principles, which focus on interconnectivity among all factors involved, and thus it reinforces the idea of a holistic approach to managing CH. Soil organic matter (SOM) content, a strong determinant of soil biodiversity and the overall soil health, is a key factor affecting soil functions and CH. Vega et al. [1] stated that CH, a multidisciplinary scientific concept, is based on the assumption that it relates to specific management and farmers’ objectives and values, focuses on human health and wellbeing, and enhances soil health and environmental attributes. Vega and colleagues proposed four components that affect CH: usefulness, adversities, safety, and autonomy. These components are pertinent to the salutogenic model for human health: meaningfulness, comprehensiveness, and manageability [1].

Therefore, the objective of this article is to describe factors affecting CH, the relation between CH and the One Health concept, methods of CH assessment, and effects of CH on human nutrition and wellbeing. Any soil health act (enacted at state, national, continental, and international level) should emphasize the necessity of managing CH while also indicating the need for research and outreach for advancing CH (to produce food, feed, fuel, and fiber); the focus should be on the wellbeing of humanity while also enhancing the health of the animals, the soil, and of the environment in which the crops are grown. In this context, protection, restoration, and sustainable management of soil health is of paramount importance. This article specifically focuses on the following issues: A) the effect of CH on human health by moderating soil health, safety, and nutritional quality of food; B) managing CH for improving human health and wellbeing by improving nutritional quality of food and forages; C) identifying key agronomic practices of managing CH; and D) finding basic strategies of enhancing CH under the overall auspices of One Health. Although the theme of CH is all-encompassing, this article is purposefully focused on a few key indicators. Readers are encouraged to search for other articles on genetic, biotic, and ranging abiotic indicators, which are beyond the scope of this article.

## The Crop–Human Health Nexus

**Fig. 2:**
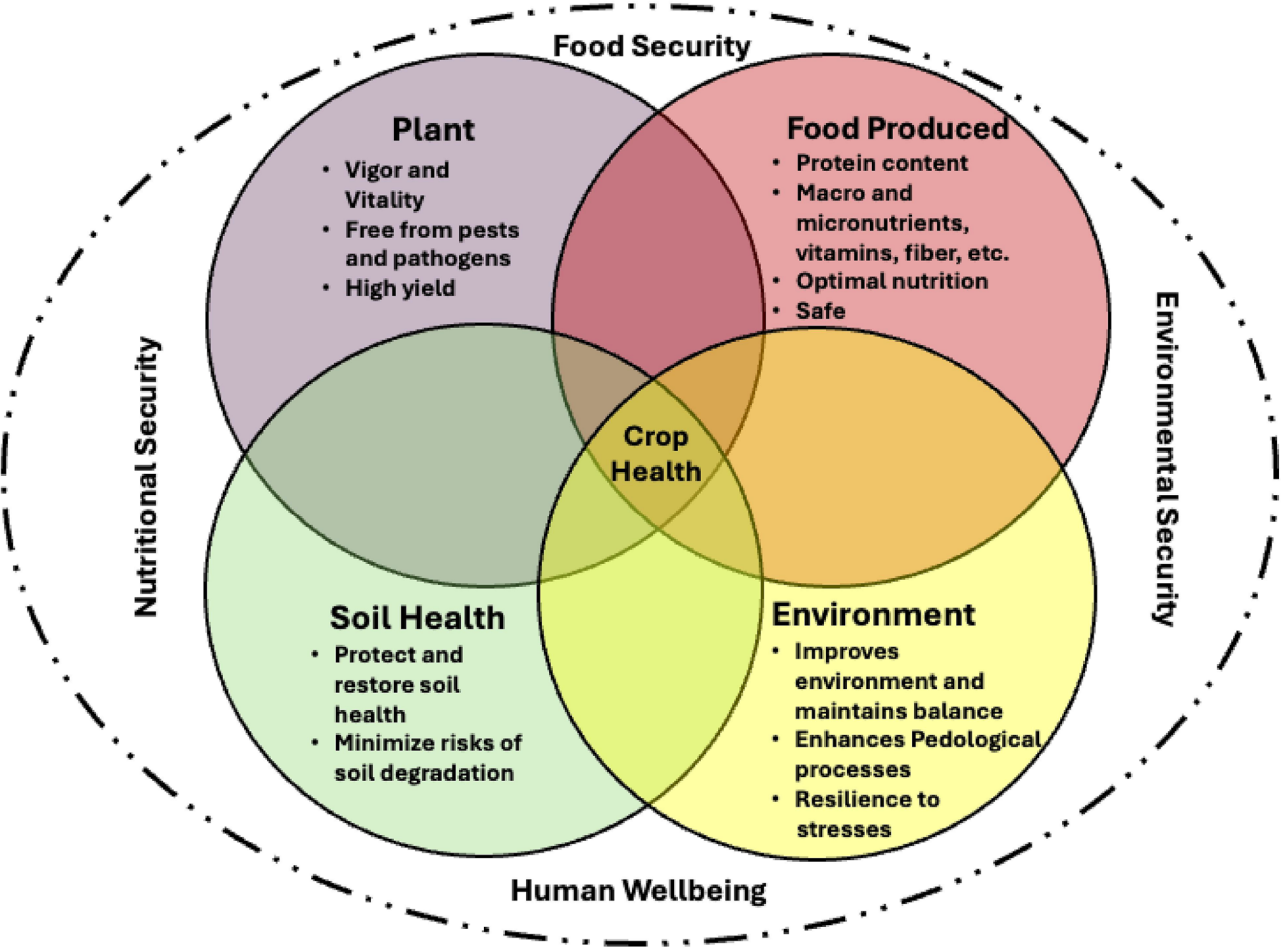
Interconnectivity of crop health management. There is a wide range of indicators of crop health, including plant, soil, nutritional quality of food, and quality of the environment. Thus, a holistic approach to crop health management is based on the interconnectivity of key determinants of plant, soil, and environment, leading to food, nutritional, and environmental security as well as human health wellbeing

The interconnectivity between CH and human health emerges from the One Health approach, which is strongly dependent on soil health. The soil health refers to its capacity to provision for ESs for human wellbeing and nature conservancy based on the optimal level of the soil’s physical, chemical, biologic, and ecologic indicators. Thus, human health and wellbeing are strongly affected by CH as moderated by soil health and its functional attributes (Fig. 2), including food safety and nutritional quality (eg, amounts of micronutrients, proteins, vitamins, fiber, etc.). The common slogan “healthy soils for healthy crops and healthy people” highlights the significance of this interconnectivity. Simply put, CH also affects human health by providing macronutrients and micronutrients and safe food that is free from harmful pathogens and compounds (agrochemicals, including fertilizers and pesticides). Food safety also depends on clean environments under which the food is grown and minimal residue of pesticides and other compounds that are used in modern agriculture.

On the basis of estimates of globally vulnerable populations, malnutrition is a bigger problem than undernutrition. Both problems are aggravated by degradation of soil health, which is a major cause of poor CH, human malnutrition, and associated health issues. Healthy soil, being the foundation of terrestrial biocommunities, must be managed judiciously because soils are crucial to all life on Earth. Healthy soil is a source of macronutrients (eg, nitrogen, phosphorus, potassium, calcium, magnesium), phytonutrients (ie, antioxidants), and 17 micronutrients that affect human health (iron, zinc, iodine, selenium, copper, molybdenum, manganese, fluoride, etc.). Deficiency of iron and iodine can lead to anemia, impaired cognitive functions, and thyroid disorders [2]. These micronutrients are contained in food grown on healthy soil. However, soils are prone to degradation by natural or anthropogenic factors, and this affects the quality of food grown on these degraded soils. Healthy soils with high SOM content can reduce the risk of incidence of mycotoxins and pathogens. With a reduced need for fungicides and insecticides, food grown on healthy soils has lower pesticide residue. The soil microbiome in healthy soil can support favorable gut microbiomes. Nutrient-dense food grown on healthy soil supports mental health and overall wellbeing. Furthermore, healthy soil has a positive impact on the environment surrounding human habitats by improving the quality of water, air, micro/mesoclimate, and above- and below-ground biodiversity. Thus, soil health indicators are linked to past management of diverse crops, including soybean [3].

**Fig. 3:**
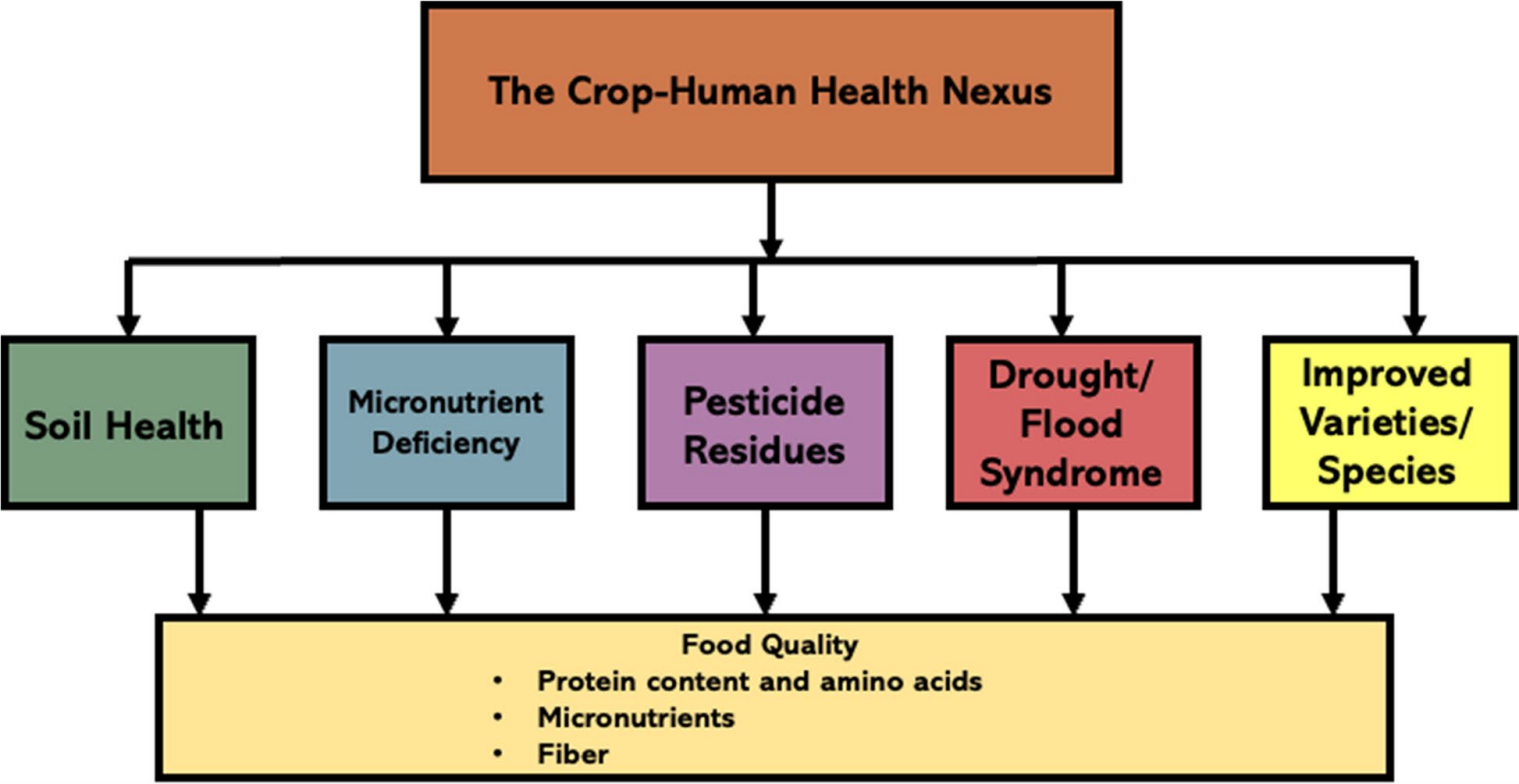
The crop–human health nexus. The crop–human health nexus is governed by soil health (micronutrients and other ingredients essential to human needs) along with other factors, such as the drought-flood syndrome, minimal residue of pesticides and other poisonous compounds, and the use of improved crop varieties, which can respond better to inputs and can be more tolerant to abiotic and biotic stresses

Because CH depends on the properties of the soil and those of the environment in which the plants are grown, its management encompasses improving soil health and optimizing the microenvironment and mesoenvironment or edaphologic conditions (Fig. 3). Soil health has four distinct but related components: physical, chemical, biologic, and ecologic. Most of these components are strongly affected by the SOM content, which in turn depends on the textural composition and minerology of the clay fraction (such as 1:1 versus 2:1 clays), regular input of biomass carbon, and judicious management of plant-available nutrients, water, and pests or pathogens. Soil clay minerals are characterized by swelling versus nonswelling attributes, which impact small versus large surface areas as well as the following factors on a low versus high scale: ion exchange capacity, aggregation and stability, aeration, risk of hydric and aeolian erosion, and root penetrability in the subsoil as determined by the pore size distribution and packing arrangement of primary and secondary particles. Low nutrient reserves, because of a low charge density and low input of nutrients, are the primary cause of low crop yields of resource-poor and small land holders of the global south. Availability of plant nutrients (inherent and applied) depends on optimal versus low capacity to hold green water supply (plant-available water) in the root zone. The coupled cycling of critical elements (ie, carbon, nitrogen, water, phosphorus, and sulfur) are activities that must not be perturbed because that leads to land misuse and soil mismanagement. A high charge density of clay minerals is also related to a high ion exchange capacity (cation and anion), which moderate retention and availability of plant nutrients and reduce losses from leaching and erosional processes. Key performance indicators (KPIs) of soil health [4] may vary among soils, but judicious management of KPIs can lead to agronomic gains from high productivity and favorable efficiency of inherent and applied water and nutrients. Biofortification is another option to enhance content of essential micronutrients in vegetables, such as planting tomatoes (see Sect. "The Crop–Human Health Nexus"C) [2]. The SOM-induced soil health, with a strong effect on human health and wellbeing (Fig. 3), can be optimally managed by a range of techniques briefly discussed below.

### Conservation agriculture

Long-term adoption of a system-based conservation agriculture (CA) can protect, restore, and sustain soil health. Beneficial effects of CA have been documented for diverse soil, crops, and ecoregions as an approach to sustainable agriculture, [5] and choice of site-specific crop rotation can enhance the positive impact of CA [6]. Use of CA in conjunction with biotechnology to improve CH is an innovative approach to enhance production of safe, nutrient-rich food and animal feed. Biotechnology crops can have a high yield, reduced pesticide use, decreased environmental damage (such as drought and salt tolerance), and other cobenefits. The micronutrient contents of crops can be increased by biofortification (see Sect. "The Crop–Human Health Nexus"C) [7]. Thus, the importance of CH in advancing global food security can never be overemphasized [8]. In this context, the Global Plant Clinic promotes international cooperation in improving CH for advancing food and nutritional security [9]. Global food security is also advanced by the use of nanoscale agrochemicals, which improve crop heath [10]. Adapting these options under site-specific situations in conjunction with CA is a win–win.

### Cover crops

Cover cropping, which is the act of planting a crop to grow in the off-season for improving soil health, has long been practiced for soil and water conservation; it also enhances nutrient availability while improving SOM content, restoring soil structure, improving soil fertility, and conserving soil by reducing risks of water runoff. Cover crops (grasses and legumes) grown as a single species or a mixture of several species, are an economic source of organic fertilizers with numerous benefits. However the usefulness of cover cropping for improving availability of plant nutrients has decreased with the increase in availability of low cost fertilizers. Nonetheless, beneficial effects of cover cropping have been widely reported through data from long-term experiments ranging from 4 to 50 years [11]. Cover cropping can also lead to environmental protection and nitrogen cycling benefits [12] while controlling soil erosion and moderating the microclimate. Improving subsoil drainage along with cover cropping can enhance the soil’s physical properties and agronomic yield. Furthermore, including a cover crop in the rotation cycle has also been shown to enhance soybean production [13] while improving soil health and enhancing the environment. Lee and McCann [14] reported that adoption of a cover crop can improve soybean production. Site-specific choice of cover crops has been documented to also increase the yield of soybean [15] and corn by improving soil parameters [16]. Therefore, cover crops are considered an important tool for sustainable agriculture in the present era of ACC and of the increasing role of organic farming. An appropriate cover crop is, in itself, a source of organic fertilizer [17]. The agroecosystem gains from cover crops are often attributed to controlling soil erosion, reducing nutrient loss by leaching, and improving soil health—these are characteristics noted in the “Corn Belt” in the heart of the United States [18].

The beneficial effects of cover cropping on soil and CH also include an increase in biodiversity. Biodiversity of cover crops can be enhanced by growing a mixture of species, which can lead to changes in root and shoot composition, decomposition of biomass, and nitrogen mineralization trends, which affect soil health and agronomic productivity [19]. Under some site-specific conditions, mixtures of cover crops may outperform monocultures in improving soil physical health [20]. However, impact of cover crops may vary among species [21], and in some cases grass species may be desirable for their decay and ability to provide substantial soil cover [21]. Crop diversity can enhance drought tolerance and reduce the environmental impact of farming [22]. Cover cropping can also improve soil fertility and plant nutrition, even in temperate climates [23]. Similar to cover cropping, agroforestry is also pertinent to advancing biodiversity and improving CH, conserving soil and water, and improving soil health [24]. Despite numerous benefits, cover crops also have some limitations and challenges [25]. Notable among these are nutrient immobilization, which leads to limited nutrition and yield, and reduction in soil water storage in the root zone for the following grain crops in arid and semi-arid regions of low water supply. In general, growing a cover crop in arid climates may aggravate drought stress because of the reduction in green water supply in the root zone.

The benefits of cover crops can also be reduced by variations in edaphic and climatic conditions. In humid environments, cover crops may increase the incidence of pests and pathogens; there can also be difficulty suppressing the cover crop growth so that they do not smother a following grain crop. Seed dispersal of an aggressive cover crop (such as *Mucuna utilis*) in the humid and subhumid tropics can climb on the succeeding corn and suppress its growth and yield. The well-known case of kudzu (*Pueraria phaseoloides*) is an indication of an inappropriate choice of a cover crop; kudzu aggressively climbs electric poles, trees, and other natural and anthropogenic structures.

### Biofortification

Deficiency of some essential micronutrients (ie, zinc, iodine, selenium, and cobalt) can affect crop and human health with severe consequences. The World Health Organization reported that annually more than 800,000 people, including around 450,000 children under the age of 5, die due to zinc deficiency [26]. Management of zinc is critical for better crop production and human health [27]. Poor human health and wellbeing affect about one billion people annually. Micronutrient deficiency can be alleviated by biofortification; this is the act of improving crops by breeding or genetic modification to improve nutritional content, which includes essential vitamins and micronutrient contents (ie, iron, zinc, vitamin A, and protein content). In addition to traditional breeding, genetic improvement can also be achieved by using biotechnology. Singh Dhaliwal et al. [28], in India, reported that zinc content in wheat can be improved by biofortification. Genetic advances in conjunction with adoption of best management practices can enhance nitrogen use efficiency and reduce leakage of nitrogen into the environment [29]; this minimizes risks of malnutrition from deficiency of some essential micronutrients [28–31]. Similar to grain crops, biofortification is also used for forage crops [28, 30].

Biofortification has been widely used to alleviate zinc deficiency in wheat and improve human health [32] and alleviate anemia due to iron deficiency [33]. Synergistic effects of biofortification in soybean–wheat rotation can be enhanced when used in conjunction with CA [34]. Biofortification and use of genetically modified crops is also an important pathway to advance food security in low-income countries [35]. In this context, improved varieties of millets have been shown to advance both food security and nutritional security [36]. This was an important reason why the United Nations declared 2023 as the “International Year of Millets.” Improving micronutrient bioavailability in crops can also be enhanced by harnessing the genetic variability among and within crops such as rice, corn, wheat, barley, soybean, and dry beans [37]. In addition to addressing genetic variability and using biotechnology, cultural practices can also be adopted that enhance uptake of micronutrients. Some examples of these practices include using fertilizer at adequate rates, finding innovative sources of fertilizer, and effective methods of fertilizer application [37].

### Enhancing organic carbon content in soil and improving soil health

**Fig. 4:**
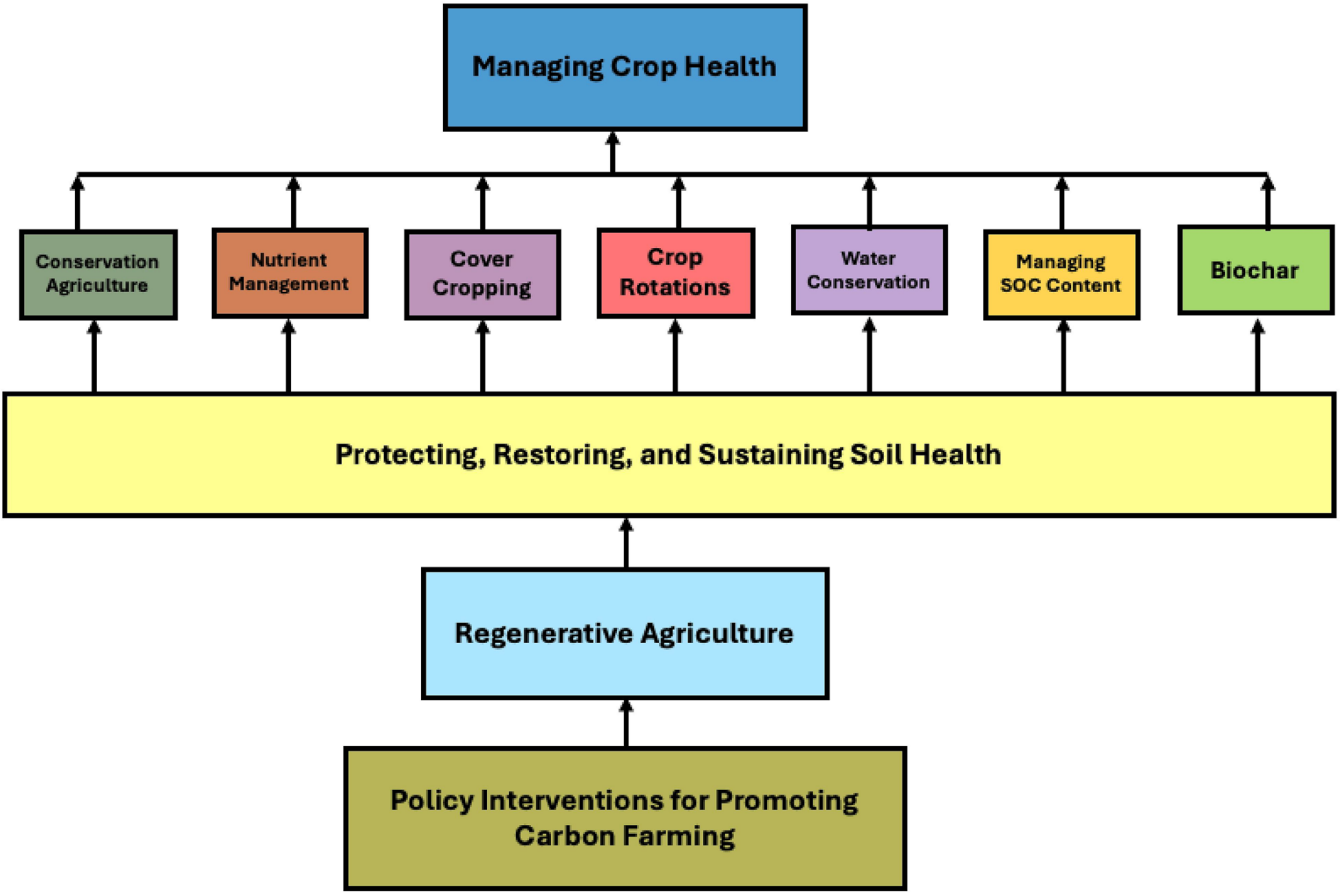
Managing crop health. Crop health can be managed by restoring soil functions through the adoption of regenerative agriculture, which has a wide range of practices; some examples are adopting system-based conservation agriculture and managing soil fertility with macronutrients and micronutrients. Managing soil fertility involves actions such as including a cover crop in the rotation cycle, increasing the availability of green water in the root zone, using amendments such as biochar, and enhancing and sustaining soil organic matter contents, which is the heart of soil health. The goal of crop management practices through the adoption of regenerative agriculture is to protect, restore, and sustain soil health through policy interventions that promote carbon farming and other emerging innovations. SOC, soil organic carbon

Soil degradation (by erosion and other processes) can lead to a rapid and strong decline in agronomic productivity, nutritional quality of food, and other ESs. Most of the zinc in animals and humans is derived from food crops grown in cultivated soils. Thus, human and animal access to zinc is strongly dependent on the nutrient presence in plants (crops and forages), which in turn is dependent on zinc levels in soil [26]. Thus, it is important to assess factors affecting availability and dynamics of zinc and other micronutrients in the soil. In this context, managing soil health by restoring soil organic carbon (SOC) content is an important strategy (Fig. 4).

Increasing SOC content in depleted and degraded soils can enhance productivity of cereals and also improve nutrient density in food. Rubio et al. [38] observed that an optimum level of SOC content was 2.0% in the surface layer, and retention-induced variation in SOC content in depleted soil can range from 1.2% to 2.6%. Celestin et al. [39] observed that site-specific choice of appropriate cropping systems can strongly impact SOC content, such as in grazed pastures versus in row-crop systems. Input of compost and manure (such as poultry manure) can also improve SOC content [40]. Adding biochar, made from pyrolysis of agricultural waste, can enhance SOC content and reduce the adverse effects of soil pollution and contamination by heavy metals [41]. Use of nanobiochar in soil can enhance SOC content [42] and the usefulness of the cropping system because nanobiochar can enhance wheat crop productivity by mitigating the effects of drought [42]. Adverse effects of recurring drought and extreme events affecting CH can be reduced by improving soil health; crop risks or failure due to dry and hot extremes [43] can be avoided by the choice of cover cropping in combination with CA. Increase in SOC content in wheat has been shown to cause an increase in nutrient content, thus making SOC management a viable tool for feeding the world well by improving small land holder agriculture [44]. In this context, restoring SOC stocks can also directly increase productivity [45]. Rather than total SOC content, soil labile carbon fractions are also considered a useful soil health indicator, especially for the potential carbon mineralizable fraction [46] under diverse farming systems, from small land holder subsistence farmers to large land holder and commercial agriculturists.

### Food security and civil strife

Political instability and wars can have adverse effects on food security, food safety, and the food’s nutrient contents. This era of political instability along with civil strife and wars are among the major factors of soil and environmental degradation and contamination of water and vegetation. Adverse effects of consuming food grown on contaminated and polluted soil can persist for generations to come with severe implications. Conflicts around the globe have already taken a heavy toll on food safety and have compromised the production of staple crops. If crops are grown in another area, away from the conflict zone, it is important to minimize risks of mycotoxin production to reduce adverse effects on health, social, and economic issues [47]. These adverse effects can be severe in the global south and other regions prone to war and civil unrest. Simply put, war pollutes water and air and degrades soil, and no one has a right or justification to destroy nature and jeopardize the health of humans and wild life for decades, even centuries, to come.

### Assessment of CH by use of artificial intelligence

There are numerous applications of artificial intelligence (AI) in the assessment of CH for on-farm conditions. Use of AI technology includes drones, sensors, and other devices for early nutrient deficiency assessment, pest and pathogen detection, and in-field soil health monitoring. These innovations lead to reduction in labor use, savings of inputs, decline in yield loss, and sustainable management of CH. Some basic applications of AI to monitor CH include precision or soil-specific agriculture, precision tillage and traffic, judicious nutrient and water management, efficient use of internal resources and external inputs, and predicting CH, vigor, and yield. Some useful technologies include machine learning, drones, Internet of Things, satellites, and mobile apps. Among the principal benefits of using AI are increased efficiency of resource use, sustainability, early intervention, and decision-making tools [48]. Other applications include modeling of CH and its impact on crop losses by plant diseases [49]. Use of AI is important for the early detection of nutrient deficiencies, diseases, and pests, allowing for timely interventions [50].

### Enhancing resilience against biotic and abiotic stresses

Improving CH also enhances resilience against biotic and abiotic stresses, such as drought. There are several types of drought that can adversely affect crop growth and agronomic productivity, such as what has been observed in Central Asia [51]. Effects of drought are aggravated by ACC, with severe adverse effects such as crop failure. In this context, the role of microbes associated with plants cannot be overemphasized [52]. In addition to advancing food and nutritional security at regional and global levels, the enhancement of crop resilience by improved soil health and advancing global food security [53] can be done by increasing farm income, addressing rural poverty, and advancing several of the sustainable development goals of the United Nations’ 2030 Agenda.

### Managing biotic and abiotic stresses to enhance CH

Good crops/plants are essential to all terrestrial life. To produce satisfactory biomass or agronomic yield, CH must be improved so that it is strong at the seedling stage and nurtured throughout the growth cycle. It is also pertinent to implement the One Health concept. Indiscriminate and overuse of pesticides may reduce crop resistance and food quality. Jia and colleagues [54] recommended the use of biostimulants (ie, microbiologic bacteria agents and nanoparticles) to sustain CH and enhance its resistance to biotic and abiotic stresses. It is also pertinent to model human health risks from pesticide use. Based on a study under Mediterranean conditions, Zemmouri et al. [55] observed that human exposure to pesticides may be higher under CA and monoculture cropping conditions than under complex farming systems. The latter involves those based on integration of crops and animals, including complex rotations based on intercropping and agroforestry. Furthermore, intercropping combined even with conventional tillage can be a sustainable option for good crop yield and human health. With the aim of implementing the One Health concept, organically grown fruits and vegetables may contain higher levels of health-promoting phytochemicals. The positive effect of organic farming may be related to lower plant stress, favorable rhizosphere microbial communities, and lower available nitrogen that can leak into the environment [56]. In general, there may be no significant differences in crop micronutrient levels between organic and conventional farming practices. However, conventional crops may contain greater pesticide levels, whereas organically grown crops may contain higher levels of phytochemicals, health-promoting antioxidants, and anti-inflammatory properties [57].

**Fig. 5:**
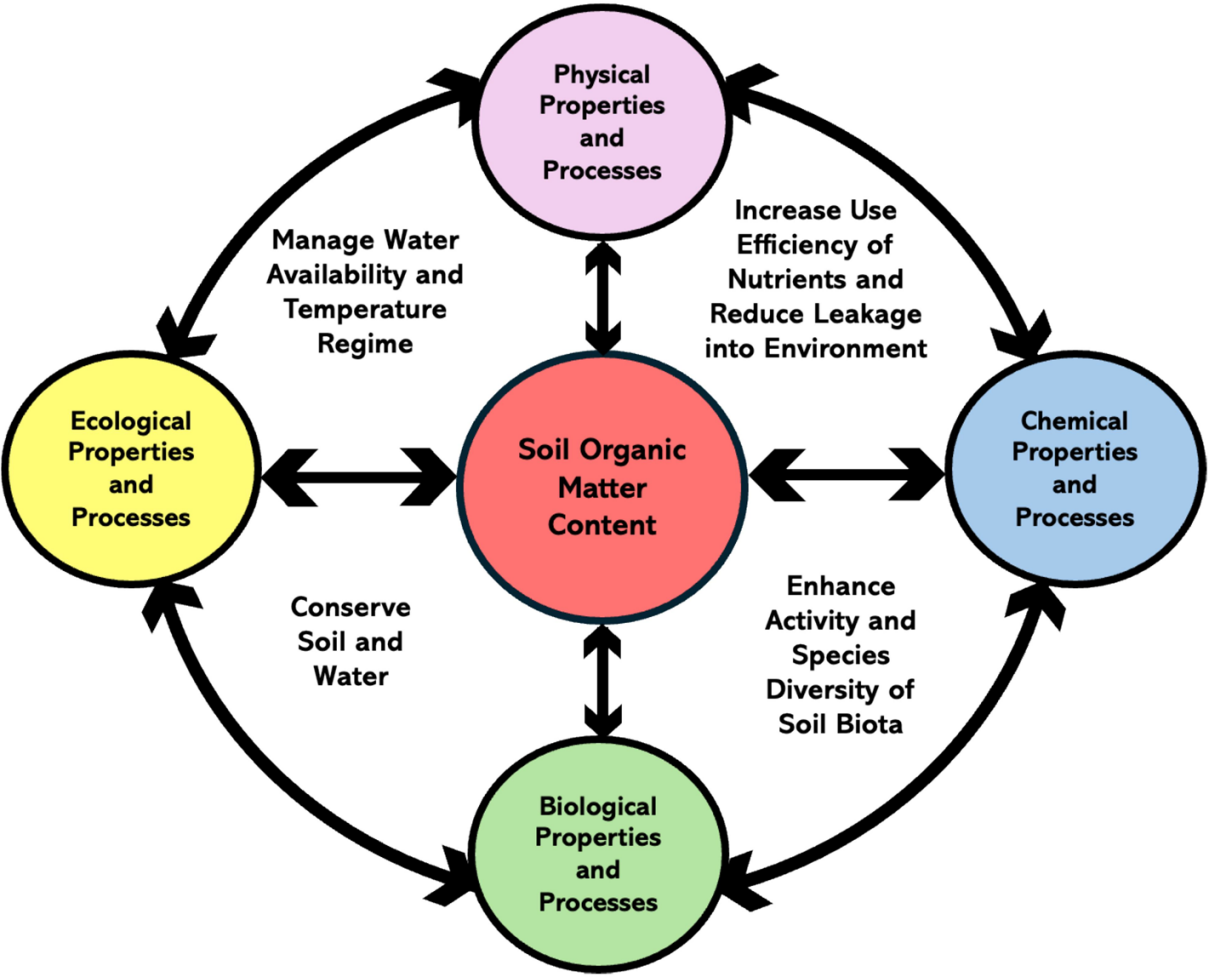
Managing soil organic matter content. Management of soil organic matter content, the heart of soil health, is critical to improving the nutritional and overall quality of crops. Soils with an optimal range of organic matter content (1.5% to 2.5% for soils in the tropics and 2.5% to 3.5% for soils in temperate climates) is also in accordance with the strategy of “producing more from less.” Healthy soils are disease-suppressive soils and thus require fewer agricultural chemicals (fertilizers and pesticides). Management of crop health needs simultaneous improvements in the physical, chemical, biologic, and ecologic health of the soil, all of which depend on the activities and species diversity of soil biota

Managing CH requires use of a holistic and systematic approach involving basic principles of agro-ecology and regenerative agriculture, with the goal of increasing above-ground (crop) and below-ground (soil) diversity (Fig. 5). The goal is to “produce more from less” so that use of inputs (chemicals) can be minimized and some land and water returned to nature.

**Fig. 6:**
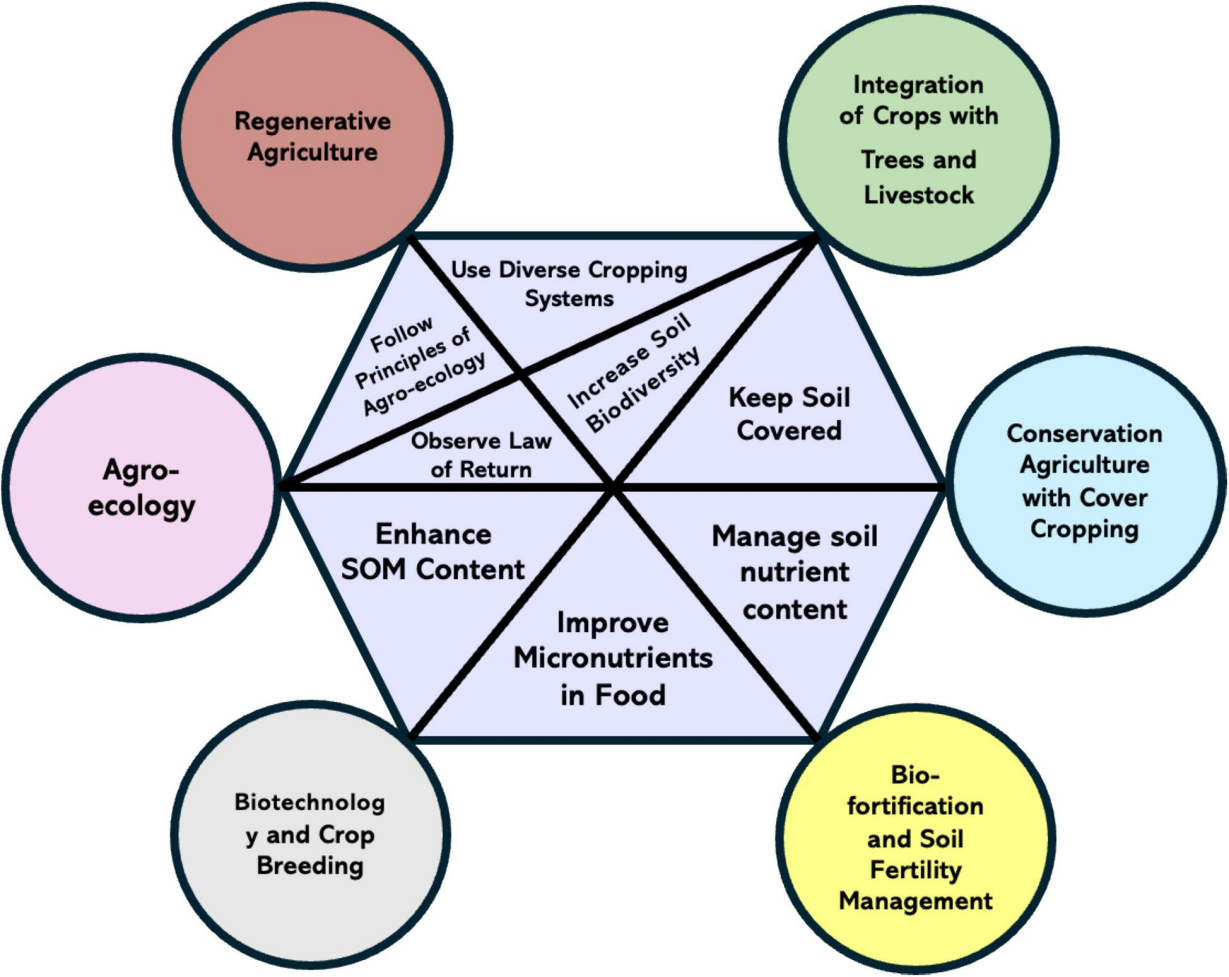
Approaches to managing crop health. Similar to managing soil health, there are diverse approaches to managing crop health. Strategies involve building the SOM content, improving the availability of macronutrients and micronutrients, keeping the soil covered with minimal disturbance, following the principles of agro-ecology, observing the “Law of Return,” and using complex farming systems based on the integration of crops and livestock. Some common practices include crop breeding, biofortification, regenerative agriculture, and system-based conservation agriculture. SOM, soil organic matter

There are diverse methods to improving CH through a holistic approach based on the principles of regenerative agriculture and agro-ecology. The interactive effects of numerous factors promoting CH are outlined in Fig. 6. Site- and ecoregion-specific combinations of developing improved CH management by crop breeding combined with regenerative agriculture and agro-ecology can benefit from adoption of CA; additionally, integrated pest and nutrient management (cover cropping, complex rotation, rhizobium for biologic nitrogen fixation, mycorrhizae) can improve soil health and make it a disease-suppressive soil for improving CH and growing safe and nutritious food. Keeping soil covered by using organic and inorganic mulches and observing the “Law of Return,” which is “replacing whatever is removed,” are of critical importance to improving CH and producing healthy, safe, nutritious food for generations to come (Fig. 6).

Carbon farming—growing carbon in land-based sinks (soils and trees) for creating another income stream—can improve human health by enhancing the health of the soil and crops. Land managers must be rewarded for provisioning and strengthening ESs through enactment of a soil health act. The private and public sector can play a strong role in improving CH and enhancing human wellbeing by protecting planetary processes. In addition to research and outreach, there is an urgent need for improving CH education at all levels. In addition to revising curricula at secondary school and college levels, early education books in local languages should be made available to primary school students so that children can learn the concepts of crop heath and the One Health approach. The private sector can also play a critical role in providing access to inputs (ie, educational material and research facilities) and translating scientific concepts into action. Humanity has a bright future ahead through education, improving curricula at all levels, and respecting the farming profession. Pro-nature, pro-agriculture, and pro-farmer policies are needed so that they can produce more from less and spare some land for nature. Preference should be given to plant-based diets, which are good for the health of humans, wildlife, and the planet. Diets that prioritize planetary health must be promoted because their impact is critical to all terrestrial life.

## Conclusion

Among the global issues of the twenty-first century are the ever-increasing problems of undernutrition and malnutrition. These challenges, driven by an increase in population—especially in low-income countries—are aggravated by poor CH, low agronomic yield, unsafe food, and deficiency in essential micronutrients and protein content. These problems are also being aggravated by ACC and the increase in the severity and extent of soil degradation. The mutually reinforcing processes of ACC and soil degradation and desertification adversely affect CH, reduce agronomic productivity, and jeopardize the nutritional quality of food. A decrease in agronomic productivity increases risks of food insecurity; a reduction in the nutritional quality of food grown on degraded soils furthers the deficiency of macronutrients, micronutrients, and other critical elements (vitamins, antioxidants, etc.). All this increases the incidence of malnutrition. Protecting, restoring, and improving CH is critical to addressing the global problems of undernutrition and malnutrition. Agronomic practices of improving CH include plant breeding, biotechnology, biofortification, cover cropping, and adoption of CA. Soil health, an important determinant of CH, is determined by its physical, chemical, biologic, and ecologic attributes. A strong determinant of soil health is its SOM content in the root zone, which for most soils should be 1.5% to 2.5% in the tropics and 2.5% to 3.5% in temperate climates. Some ways to manage SOM content to enhance CH are adoption of CA, cover cropping, and integrated nutrient management based on a judicious combination of organic and inorganic amendments. Maintaining SOM content in the root zone enhances activity and species diversity of soil biota, creates disease-suppressive soils, and also improves the soil structure, its plant available water, and nutrient holding capacity. These parameters in turn improve soil health and the nutritional quality of food. Creation of disease-suppressive soils reduces the use of pesticides and thus decreases the risk of food contamination from chemical residue.

Regenerative agriculture and agro-ecology use innovative approaches to restore and enhance soil health, which improves CH. One of these approaches is carbon farming, or growing carbon in soil as a commodity, which can create another income stream for farmers. Farming carbon, similar to CA and cover cropping, also increases SOM content and improves CH. Carbon farming can also lead to another income stream for land managers. Enactment of soil health acts at local, state, national, continental, and international levels is important to promoting carbon farming through payments for ESs. Agricultural education is important to build understanding of planetary processes that are driven by the lifestyle of humanity and the changing demands of a rapidly growing and increasingly affluent human population.

Some examples of researchable priorities include the following: A) multidisciplinary research on the application of the One Health concept under on-farm conditions, with specific focus on strengthening the interconnected factors affecting CH; B) identification of crop-specific indicators of CH and their management; C) determining practical techniques of improving micronutrients and protein content in relation to CH; D) promotion of emerging and innovative ideas that improve CH, such as agro-ecology, carbon farming, and subsurface drip fertigation; E) breeding of crop varieties that emit molecular-based signals under diverse biotic and abiotic stresses, plus those which can be detected remotely; F) involving farmers and extension agents for promoting CH in the context of combatting hidden hunger; G) interacting with policy makers to promote the nexus of crop, soil, environment, and human health; H) revising educational curricula to include CH in relation to soil and environmental health in primary and secondary schools; I) rewarding farmers for adopting agro-ecology and regenerative agriculture that enhance and improve CH; and J) developing indicators of CH that scientists can quantify and farmers can also understand and apply in their regular work.

## Data Availability

This is a review article, and no original data are involved.
